# Can blood serum amyloid A concentrations in horses differentiate synovial sepsis from extrasynovial inflammation and determine response to treatment?

**DOI:** 10.1136/vr.105153

**Published:** 2020-09-17

**Authors:** Matthew Sinovich, Nicolas F Villarino, Ellen Singer, Claire S Robinson, Luis M Rubio-Martínez

**Affiliations:** 1 Department of Equine Clinical Science, Institute of Veterinary Science, University of Liverpool, Neston, Cheshire, UK; 2 Program in Individualised Medicine, Washington State University, Washington, DC, USA; 3 E Singer Equine Surgery and Orthopaedics, Parkgate, UK; 4 Nantwich Veterinary Group, Nantwich, UK; 5 Sussex Equine Hospital, Ashington, Sussex, UK

**Keywords:** horse, sepsis, SAA, serum

## Abstract

**Background:**

Serum amyloid A (SAA) concentrations in blood and synovial fluid of horses with synovial sepsis have diagnostic value. Studies suggest serial blood SAA measurements could act as a prognostic indicator. This study evaluated the use of serial blood SAA concentrations for monitoring of horses with synovial sepsis.

**Methods:**

A prospective clinical trial was performed of horses referred to a single hospital with synovial sepsis that survived (n=17), synovial sepsis that were euthanised (n=5), non-septic intrasynovial pathologies (n=14) or extensive extrasynovial lacerations (n=5). SAA concentrations were determined on admission and every 24 hours thereafter. The area under the concentration–time curve from 0 to 144 hours of each group was compared by Kruskal-Wallis and post hoc Dunn’s tests (P<0.05).

**Results:**

Significant difference in mean blood concentration of SAA was found between synovial sepsis that survived and non-septic pathologies in the first 48 hours, as well as between non-septic intrasynovial pathologies and non-responsive sepsis requiring euthanasia. No difference was found between extensive extrasynovial lacerations and any septic group.

**Conclusions:**

While serial blood SAA is useful for monitoring clinical response of intrasynovial septic pathologies, interpretation should consider other clinical findings since blood SAA is not a specific marker for synovial sepsis.

## Introduction

Serum amyloid A (SAA), one of the main acute phase proteins in horses,^1–3^ has been shown to be a differentiating factor for septic and non-septic inflammatory conditions including synovitis.^2 4–8^ When a horse acquires an extensive laceration or synovial contamination, the introduction of foreign material and pathogens stimulates a marked inflammatory response^2 9^ with local swelling, pain and heat. In synovial structures, this response will eventually lead to the enzymatic breakdown of hyaluronic acid and cartilage.^9 10^ The local release of cytokines such as interleukin- 1 (IL-1), tumour necrosis factor-alpha (TNF-α) and interleukin-6 (IL-6) from monocytes and macrophages at the site of injury drives the inflammatory response. Once released into the bloodstream, these pro-inflammatory cytokines stimulate synthesis of acute-phase proteins such as SAA by the liver.^2 3^

Monitoring response to treatment in horses with synovial sepsis has traditionally relied on white blood cell count and total protein determination in synovial fluid (SF) after repeated synoviocentesis.^11^ However, those SF parameters are affected by routine synoviocentesis, administration of intrasynovial medication and surgical synovial treatments, which makes their clinical interpretation difficult in some cases.^2 7 12–16^ The intrasynovial procedures listed seem to have a limited effect on blood SAA, which has been proposed as a potential parameter to monitor therapy and resolution of synovial sepsis.

Serum concentrations of SAA have been shown to peak 48 hours after the onset of inflammation.^2 8^ SAA can undergo a several thousand-fold increase from baseline values, followed by a rapid decrease in concentration once the infectious or inflammatory stimulus has been removed.^2 3 8^ A recent study compared SAA concentrations in blood and SF of horses with synovial sepsis to horses with non-septic synovial pathologies.^12^ Both blood and SF concentrations of SAA had diagnostic value in horses suffering from synovial sepsis.^12^ Another study monitored blood SAA values every 48 hours in horses undergoing treatment for synovial sepsis^2^ and agreed with these findings. These studies have suggested that serial measurements of SAA in horses with synovial sepsis could guide treatment and act as a prognostic indicator for survival.^2 12^

Systemic inflammatory and infectious conditions can also increase blood SAA concentrations,^1^ which if concurrent with septic synovitis could confound interpretation of results and limit the diagnostic or monitoring potential of SAA concentrations. Many cases of synovial sepsis occur as sequelae to significant wounds or trauma causing contamination of synovial cavities. If synovial sepsis is present in conjunction with a significant wound, peri-articular inflammation and/or infection could lead to increased systemic SAA concentration which would confound the interpretation of SAA values in relation to synovial sepsis.

Horses with non-septic intrasynovial pathology have been reported to have low synovial and serum SAA concentrations.^12 17^ While SAA concentrations in blood and SF did not increase after repeated intra-articular injection of antimicrobials or after arthroscopic or needle articular lavage,^13–15^ certain intrasynovial surgical techniques may involve significant tissue trauma that might increase systemic SAA.^1^ The effect of extensive extrasynovial lacerations or intrasynovial surgery on SAA has not been investigated or compared with values of SAA of horses with septic synovial structures.

The aim of this study was to compare the serial SAA concentrations in the blood of horses suffering from synovial sepsis with horses with non-septic intrasynovial pathologies and horses with extensive extrasynovial lacerations. Serial blood concentrations of SAA were compared in horses undergoing surgical treatment for synovial sepsis that survived, synovial sepsis that were euthanised, non-septic intrasynovial pathologies or extensive extrasynovial lacerations that underwent surgical debridement and repair under general anaesthesia. The authors hypothesised that serial blood SAA concentrations in horses with septic synovitis subjected to endoscopic treatment under general anaesthesia would be higher than in horses with non-septic intrasynovial pathology or extensive extrasynovial lacerations that underwent surgical treatment. The second hypothesis was that serial blood SAA concentrations in horses with septic synovitis which responded favourably to surgical therapy would be lower and decline faster than in those with synovial sepsis, which did not respond to treatment.

## Materials and methods

### Inclusion criteria

For this clinical longitudinal observational study, performed between May 2015 and August 2016, inclusion criteria were horses presented to a single equine referral hospital for surgical treatment of synovial sepsis, non-septic intrasynovial pathology or a significant laceration without the involvement of synovial structures which required wound debridement and reconstruction under general anaesthesia.

### Study groups

Horses were included in one of four groups: group 1—horses with confirmed synovial contamination or sepsis (S-SS), which were responsive to intrasynovial endoscopic treatment; group 2—horses with non-septic intrasynovial pathology (S-NS) which underwent intrasynovial endoscopic surgery; group 3—horses with confirmed synovial sepsis (S-SE), which failed to respond to surgical endoscopic treatment and were euthanised; group 4—horses with extensive lacerations, which did not involve synovial structures and underwent surgical wound debridement and reconstruction (ES-L). All horses underwent general anaesthesia for the required procedures.

Horses were included in the S-SS group if they met one or more of the following criteria^12^: synovial fluid parameters consistent with synovial sepsis (total nucleated cell count >20×10^9^ cells/L, total neutrophil percentage over 80 per cent and a total protein over 30 g/L); direct communication of a wound with a synovial cavity established visually, endoscopically or visualisation of fluid from joint pressure test; positive bacterial culture from synovial fluid or membrane sample; presence of intracellular bacteria on a synovial fluid smear. Horses were included in the S-NS group if they underwent endoscopy for intrasynovial conditions including osteoarthritis, osteochondrosis, intra-articular fracture and non-septic tenosynovitis. Horses were included in the S-SE group if they met the criteria for synovial sepsis as for the SS-S group, showed no clinical response to endoscopic lavage and were euthanised.

Horses were included in the ES-L group if they had a laceration, which did not involve a synovial structure but required surgical debridement and repair under general anaesthesia. Horses with other concurrent systemic inflammatory conditions were not included in the study.

### Sample collection and processing

Jugular blood samples were collected in plain tubes with clot activator (BD Vacutainer, Belliver Industrial Estate, Plymouth, UK) from the horses on the day of admission prior to anaesthesia and then every 24 hours until discharge or euthanasia. Samples were centrifuged within 10 min of collection at 4000 *g* for 15 min at 4°C. Serum was removed and stored at –80°C until analysis was performed as previously described.^12^

### SAA quantification

All sample analysis was performed by a single operator to limit variability.^12^ The samples were removed from the freezer, thawed at room temperature and then processed immediately. The SAA concentration was determined with an automated chemistry analyser (IDEXX Catalyst One; IDEXX Laboratories Limited, St. Helens, Merseyside, UK) with a human SAA turbidometric immunoassay (Eiken LZ SAA test; Eiken Chemical Mast group, Bootle, Merseyside, UK) previously validated for use on equine samples.^7 12 15 18^ A zero value was assigned to any concentrations which were below the limit of detection which was 0.5 µg/ml.^12^

### Statistical analysis

Descriptive statistics were performed and where appropriate data are presented as median (range). Blood SAA concentrations were plotted versus the time points and the area under concentration versus time curve from 0 to 144 hours was calculated by the trapezoid rule.^19^ The AUC was not normally distributed (Shapiro-Wilk test P>0.05). The median AUC for each group was compared using Kruskal-Wallis test and post hoc Dunn’s test was then performed. Statistical analysis was performed using R studio V.3.1. The significance level was set at P value <0.05.

## Results

Forty-one horses were included in the study: S-SS (n=17), S-NS (n=14), S-SE (n=5) and NS-L (n=5). The study groups included 21 geldings and 14 mares. Horses included one Appaloosa, three Arabians, three Irish Cobs, one Connemara, four Irish Draft crosses, six Irish Sport horses, one pony, one Shire cross, six Thoroughbreds, four Thoroughbred crosses, four Warmbloods and five Welsh horses. The median age of horses was 12 years (range, 2–19 years). Distributions of structures affected are listed in table 1. The aetiology of sepsis included sepsis post intrasynovial medication,^1^ puncture wounds^10^ and traumatic wounds and lacerations.^11^

**Table 1:** Synovial structures affected in the horses included in the study

Structure affected	Horses (n)
Digital flexor tendon sheath	9
Metacarpophalangeal/metatarsophalangeal joint	7
Tarsocrural joint	3
Radiocarpal joint	5
Navicular bursa	2
Distal interphalangeal joint	6
Calcanean bursa	2
Scapulohumeral joint	1

**Figure 1:**
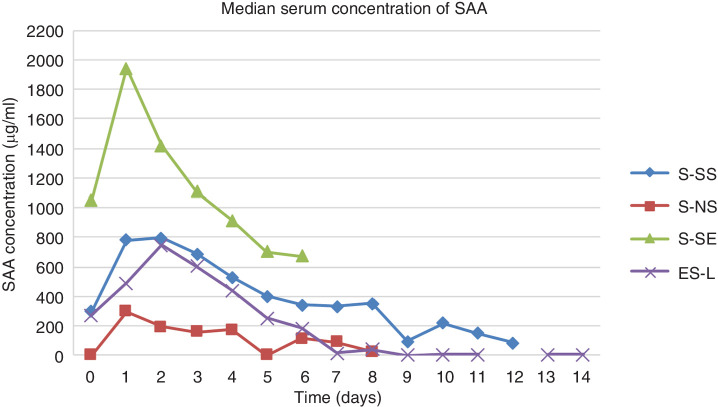
Median concentration versus time of blood serum amyloid A (SAA) in horses included in the study. ES-L (n=5), extrasynovial lacerations; S-NS (n=14), non-septic intrasynovial; S-SE (n=5), synovial sepsis—euthanised; S-SS (n=17), synovial sepsis—survived. Time zero indicates the day of admission.

**Figure 2:**
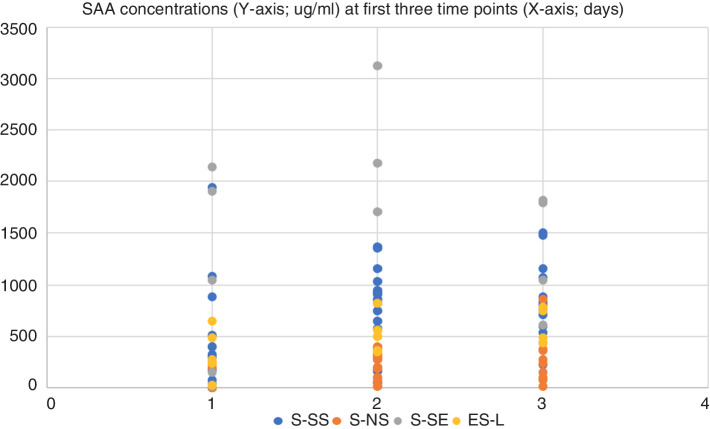
Scatter plot of blood serum amyloid A (SAA) values for all horses. The X-axis shows the first three time points (days) and the Y-axis shows the distribution of values (µg/ml). ES-L, extrasynovial lacerations; S-NS, non-septic intrasynovial; S-SE, synovial sepsis—euthanised; S-SS, synovial sepsis—survived.

The temporal kinetics of SAA blood concentrations in horses included in the study are shown in figure 1. A scatterplot of the blood SAA values for the first three time points has been included as figure 2 to show the distribution of values among groups. The S-SS group had a median blood concentration of SAA of 296.2 µg/ml on admission that peaked at 796 µg/ml after day 2 and then tapered off over the following time points (table 2). Concentrations of blood SAA in S-SS group were significantly higher than the S-NS group at admission, day 1 and day 2.

**Table 2:** Median concentrations of SAA per group at admission and over the first 4 days

Groups	Blood SAA (µg/ml) concentration for time points (days)					Median AUC (µg*h/ml)
	Days	1	2	3	4	
S-SS	Median	296	780	796	687	59 933
	Range	9–1939	55–1359	220–1510	145–1103	
S-NS	Median	4	300	192	159	9082
	Range	0–245	9–368	6–380	12–862	
S-SE	Median	1049	1942	1419	1110	152 942
	Range	8–2139	363–3130	607–1791	611–1110	
ES-L	Median	275	492	749	606	63 701
	Range	24–642	350–817	433–894	464–692	

Median AUC is shown for group in the final column.

ES-L, extrasynovial laceration; SAA, serum amyloid A; S-NS, synovial non-septic; S-SE, synovial septic euthanased; S-SS, synovial septic survived.

The S-NS group had significantly lower median blood concentrations of SAA than all other groups for the first 4 days. Blood concentration of SAA in the S-NS group was significantly lower than all other groups at admission. Blood SAA was also significantly lower than both the S-SS and S-SE group at days 1 and 2. At day 3, SAA in S-NS group was still significantly lower than the S-SE group. Significant P values are summarised in table 3.

**Table 3:** Summary of significant P values from Dunn’s comparisons of median SAA concentrations at the first four time points

Significant P values from Dunn’s comparisons of median blood SAA concentrations (µg/ml) at different time points (days) for different groups					
Groups	0	1	2	3	4
S-SS vs S-NS	0.0002	0.0009	0.0007	X	X
S-SS vs S-SE	X	X	X	X	X
S-SS vs ES-L	X	X	X	X	X
S-NS vs S-SE	0.0002	0.0009	0.0007	0.0082	X
S-NS vs ES-L	0.0002	X	X	X	X
S-SE vs ES-L	X	X	X	X	X

X indicates absence of statistical significance (P>0.05).

ES-L, extrasynovial lacerations; SAA, serum amyloid A; S-NS, synovial non-septic; S-SE, synovial septic euthanased; S-SS, synovial septic survived.

Some of the highest median blood SAA concentrations on admission were obtained in the S-SE group (1049 µg/ml; range, 8.7–2139.1 µg/ml). The median increased at day 1 and then decreased gradually. Blood SSA in S-SE was significantly different to the S-NS group at admission as well as at days 1, 2 and 3.

The ES-L group had a median SAA value on admission of 275 µg/ml which was similar to the SS-S group but significantly elevated relative to the S-NS group, with no difference compared with the S-SE group. The blood SAA level of the ES-L group increased to a peak at day 3 and then decreased. There were no significant differences between the ES-L group and any other group at any other time point.

The AUC of blood SAA in S-NS was significantly lower than both the S-SS and S-SE (P=0.0001) groups. The AUC of the S-SE groups was significantly higher than the S-NS group (P<0.0001) but not significantly different compared with the other groups. The AUC of the ES-L group was also not significantly different to any group.

## Discussion

In this study, blood concentrations of SAA were monitored daily in horses undergoing surgical treatment for synovial sepsis, synovial conditions other than sepsis and extrasynovial lacerations. This study has shown that serial blood concentrations of SAA are useful as indicators of response to treatment in horses with synovial sepsis or contamination following treatment; however, concurrent conditions other than synovial sepsis can produce a significant inflammatory stimulus leading to increased blood SAA values which can be confounding.

Previous studies have shown that blood SAA levels in horses with septic synovial structures should decrease with a positive response to treatment.^1 2^ These findings were supported by the current study with blood SAA concentrations in the S-SS similar to those in the S-NS group by day 3 post-surgery. Horses with synovial sepsis that did not respond to treatment had higher blood SAA values initially with a decrease after surgical treatment; however, at 4 days, values remained above 600 µg/ml. Unfortunately, the variability of SAA values and the limited number of cases included in this study did not allow the authors to define cut-off values between responding and non-responding cases. Similar variability in SAA values has been reported in other studies^2 12 20^ and may reflect different severity of inflammatory stimulus, bacterial load and/or virulence.^1 9^ Despite the lack of a clear cut-off value, serial SAA measurements can assist with clinical decision-making and the trend in values may be used in conjunction with other clinical parameters to determine response to treatment.

Blood concentrations of SAA have been shown to take 4–8 hours to elevate in a lipopolysaccharide-induced arthritis model in equines^18^ and have been reported to peak at 36 and 48 hours after experimental synovial inflammation and sepsis^20^ and synovial penetration injury,^2^ respectively. The chronicity of the insult was not recorded in the current study; however, in the reported population, the majority of cases of synovial sepsis and contamination are secondary to wounds and typically present to the hospital more than 6–8 hours after the injury occurred. Similar to previous studies, horses responding to surgical intervention showed blood SAA falling to 200–300 µg/ml before 7–8 days.^2 20^ Horses with synovial sepsis unresponsive to treatment showed the highest blood SAA concentrations throughout the study time, with values at the time of euthanasia within the range of 600–800 µg/ml, although these were not statistically different from the groups of sepsis that survived and significant lacerations. Production of SAA is related to the severity of the inflammatory stimulus,^21^ which is determined by the degree of contamination, tissue damage, bacterial load and bacterial virulence.^1 9^ Previous studies have reported high levels of SAA following complications after both elective and emergency surgeries in horses, which remained persistently high until resolution of the complications.^2 17 22^ Further studies are necessary to investigate potential association between SAA concentration and number of surgical procedures required and to further investigate the suggested association between SAA concentration and prognosis.

Synovial contamination and subsequent sepsis is a common sequela in horses following penetrating injury and remains a frequent occurrence in equine practice.^2 10 23 24^ This study shows that extensive extrasynovial pathology can represent a systemic inflammatory stimulus of similar magnitude to synovial sepsis and cause similar increased blood SAA concentrations. The reduction in blood SAA in the S-SS group should be interpreted as a resolution of infection and inflammation in both the intrasynovial and extrasynovial tissues. Elevation of blood SAA has also been recognised following castration in horses and median blood SAA in horses with castration site infection were 600–700 µg/ml for at least 8 days postoperatively, which are similar to horses with synovial sepsis that responded to treatment and horses with extrasynovial lacerations in this study.^12 17^ When interpreting blood SAA concentrations in horses treated for synovial infection, the contribution of extrasynovial inflammation should be carefully considered as blood SAA is not a specific marker for synovial sepsis.

Surgical trauma can cause increased blood SAA concentrations and the elevation is related to the degree of surgical trauma.^1^ In the S-NS group, horses that underwent arthroscopic surgical trauma had up to a hundredfold increase in median blood SAA concentration after surgery. Peak concentrations were recorded 48 hours (>300 µg/ml) after the surgical stimulus and then rapidly tapered off over the following 48 hours. The curve of the peak concentration of blood SAA in the S-NS group remained lower than the ES-L group and the curve decreased more slowly, but there was no statistical difference after 24 hours. This suggests that although surgical trauma creates a marked stimulus for SAA production, it is below the intensity of a significant laceration with tissue inflammation and infection. The median blood SAA values after the peak seemed to decrease in a similar manner between the ES-L, S-NS and S-SS groups. The degree of elevation of blood SAA seen following endoscopic treatment was significantly higher (three times higher) than anticipated and is in contrast with previous literature.

Synovial fluid concentrations of SAA were not monitored since synoviocentesis on a routine basis cannot be justified in horses without synovial sepsis. Previous studies have shown moderate correlations between concentrations of SAA in blood and synovial fluid in horses, with higher blood SAA concentrations compared with synovial concentrations in cases of synovial sepsis.^12^ Although synoviocytes can produce SAA in the face of synovial inflammation or sepsis,^18^ an extra-articular source of synovial SAA has been suggested in horses with synovial sepsis.^12^ In fact, systemically derived isoforms of SAA have been isolated in the synovial fluid of humans and horses^7 25^ and the liver is considered as the main source of SAA in the horse.^18^

Horses in this study with non-responsive synovial sepsis or contamination had the highest SAA values on admission and over the first 36 hours of hospitalisation. These high values could be related to a more serious infection, more virulent bacteria or longer time to referral, incomplete or insufficient lavage and/or debridement, foreign material, features which were not within the scope of this study. The lack of significant difference in the AUC in S-SE group compared with other groups with a favourable outcome may be due to the fact that 40% (2/5) of the horses in the S-SE group were euthanised earlier based on the lack of clinical improvement, with fewer serial blood samples obtained and therefore, limited AUC values. This may also be a lack of power due the number of horses included.

The majority of cases had received some NSAIDs prior to referral or during treatment. The role of NSAIDs on the response of acute phase proteins has yet to be evaluated in the horse. Studies in humans, dogs and ruminants provide conflicting evidence regarding the effect of NSAIDs on acute phase protein concentrations, with some studies showing that NSAIDs administration did not affect acute phase protein response in comparison with non-treated controls^26 27^ and other studies demonstrating the opposite.^28 29^

Limitations in the current study include the limited number of horses included and the fact that the number of blood samples differed among groups as was dependent on response to treatment, resolution of the disease or euthanasia. Due to the clinical nature of this study, factors such as synovial structures affected, synovial conditions, anatomical locations of lacerations, inciting causes of sepsis and time from injury to initial sample could not be controlled and are suspected to have contributed to variability in SAA data. Investigation of SAA response to synovial sepsis without concurrent soft-tissue inflammation or infection may be warranted to help define cut-off values which would aid with clinical prognosis.

## Conclusion

Blood SAA is not a specific marker for synovial sepsis in horses and interpretation of blood SAA concentrations should be performed in conjunction with other clinical findings. Serial monitoring of blood SAA in horses may be useful for monitoring clinical response of septic synovial pathologies; however, it is important to consider the effect on SAA concentrations of concurrent confounding pathologies such as significant lacerations.
